# Supplementary material for: Combined Metabolome and Transcriptome Analysis Elucidates Sugar Accumulation in Wucai (Brassica campestris L.)
Source: Int J Mol Sci. 2023 Mar 2;24(5):4816. doi: 10.3390/ijms24054816 (PMC10003340; doi:10.3390/ijms24054816)
Supplement: Supplementary file 1 [file ijms-24-04816-s001.zip › ijms-2104373-supplementary.pdf]

International Journal of
Molecular Sciences

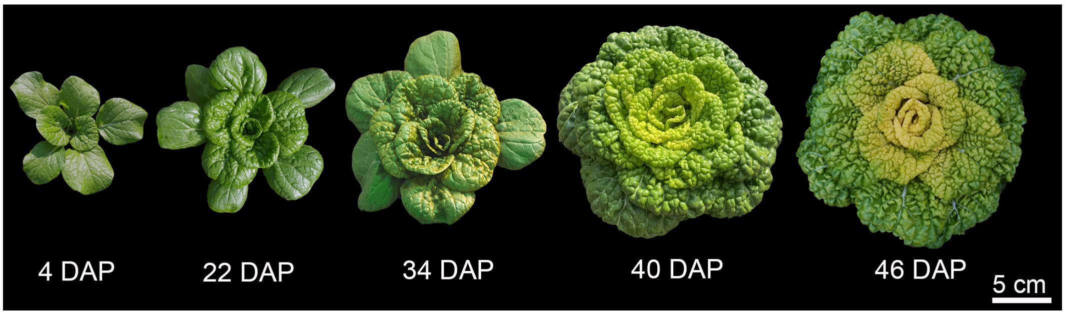

Supplementary Figure S1. Pictures of wucai used in this study. The wucai leaves at 34 DAP and 46 DAP were subjected to metabolome and transcriptome analysis. Samples (vein removed) were taken at 8.30 am.

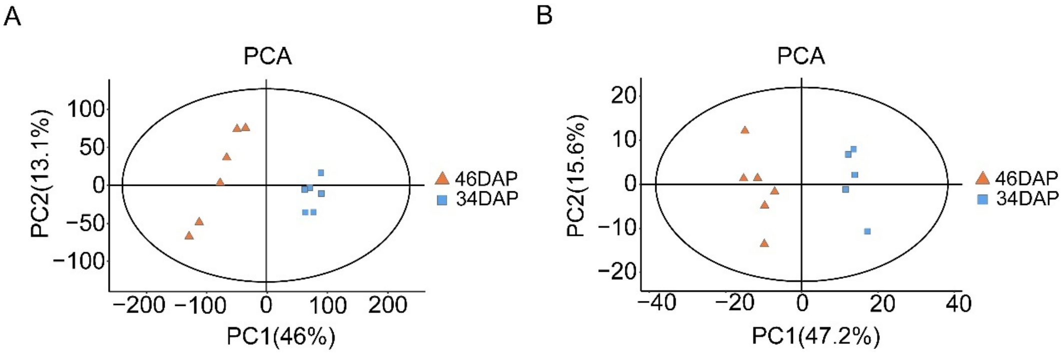

Supplementary Figure S2. Principal component analysis based on LC-MS/MS and GC-MS/MS in wucai leaves. (A) LC-MS/MS. (B) GC-MS/MS. The abscissa and ordinate represent the score value projected on the principal components PC1 and PC2 of each sample, respectively. The position of the sample reflects the difference between other samples. One sample collected at 34 DAP in GC-MS/MS had poor repeatability and therefore its information was deleted.

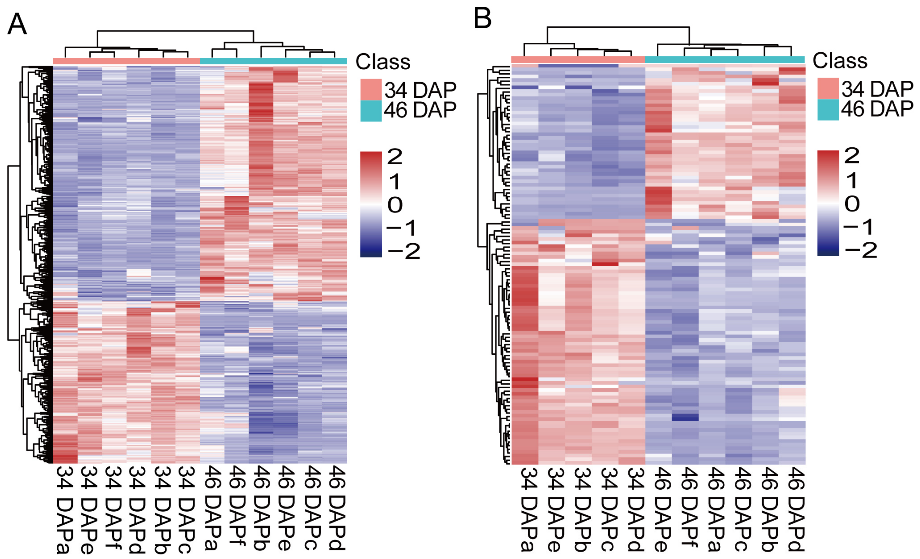

Supplementary Figure S3. Heat map of DAMs in LC-MS/MS and GC-MS/MS. (A) LC-MS/MS. (B) GC-MS/MS. The data obtained from expression abundance of DAMs.

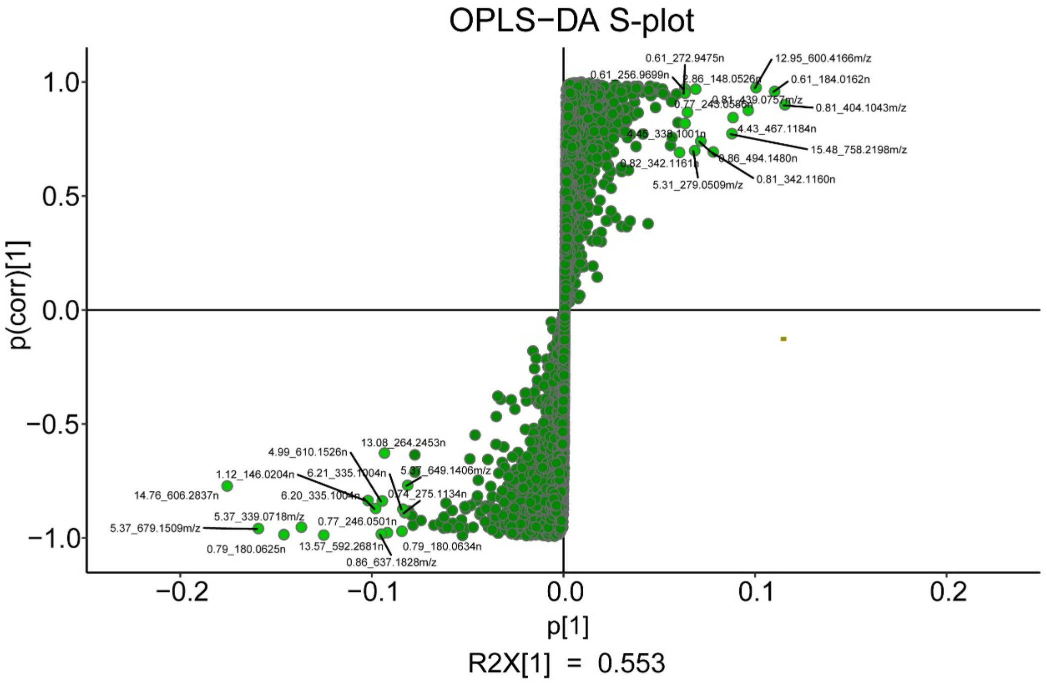

Supplementary Figure S4. OPLS-DA S-plot in LC-MS/MS. The x-axis is the eigenvalue of the influence of metabolites on the comparison group, and the y-axis is the correlation between sample score and metabolites. Figures marked at the top right and bottom left represent the IDs of the DAMs.

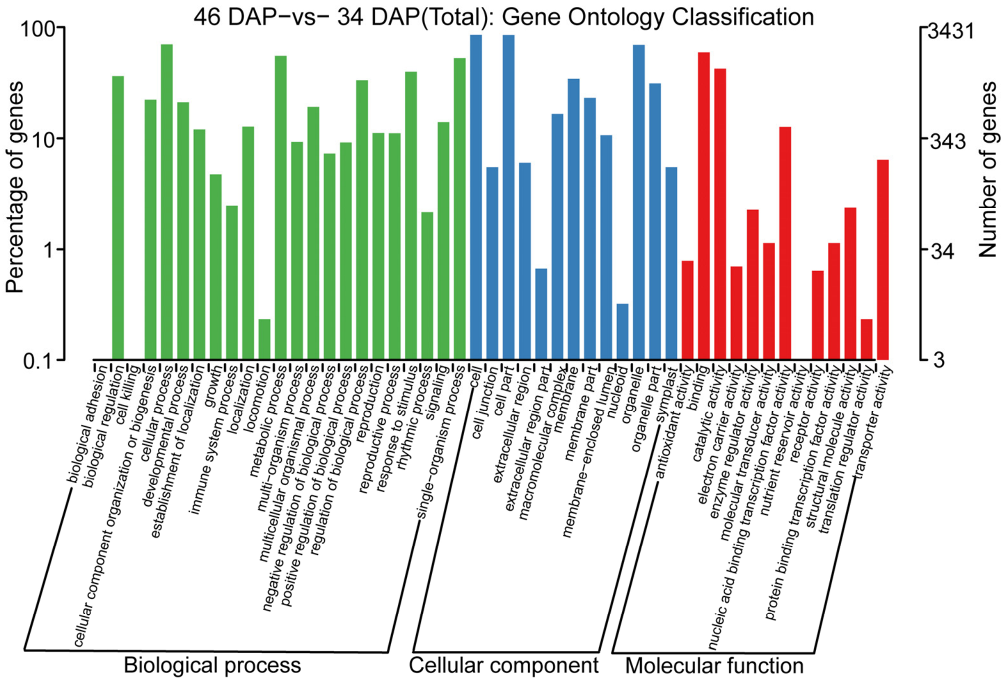

Supplementary Figure S5. GO distribution of DEGs in wucai leaves at 34 DAP and 46 DAP. The DEGs were based on GO categories that were grouped into three levels: biological process, cellular component, and molecular function, the left and right y-axis shows the percentage and number of genes, and the x-axis indicates specific categories of genes.

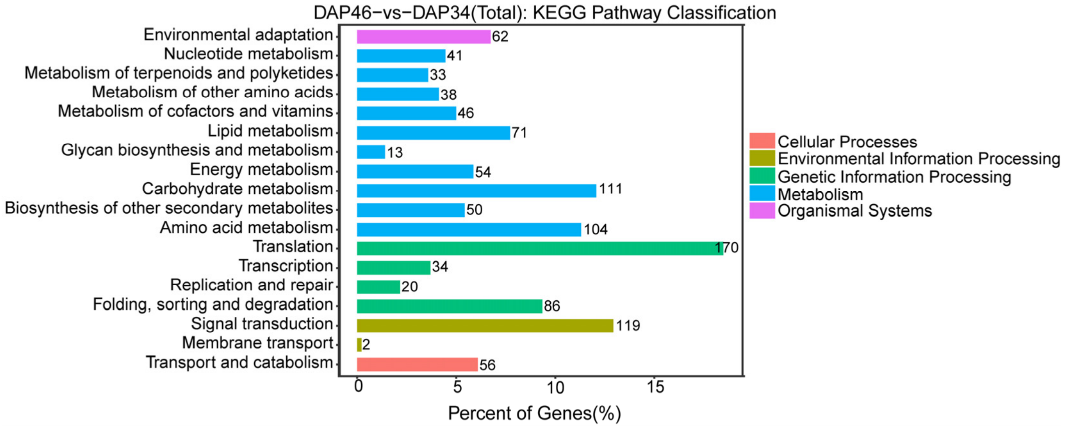

Supplementary Figure S6. Unigenes of wucai leaves annotated in the KEGG database. The DEGs were based on KEGG categories that were grouped into five levels: cellular processes, environmental information processing, genetic information processing, metabolism, and organismal systems. The left axis shows specific categories of the genes. The x-axis indicates percentage of genes.

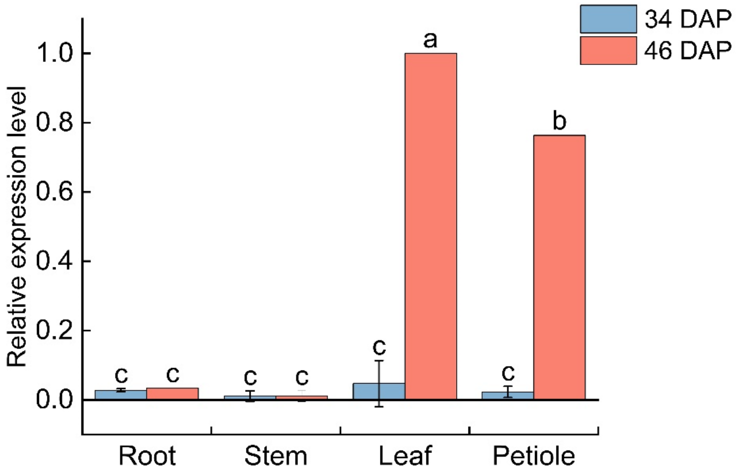

Supplementary Figure S7. Relative expression levels of CWINV4. Relative expression levels in wucai root, stem, leaf, and petiole at 34 DAP and 46 DAP. The relative expression in the leaves at 46 DAP was normalized to 1. Bars with different letters are significantly different at $P < 0.05$.

Supplementary Table S1 Classification analysis of DAMs in LC-MS/MS

	Category	Number of metabolites	Proportion (%)
Sup Class	Alkaloids and derivatives	2	0.31%
	Benzenoids	19	2.92%
	Hydrocarbons	1	0.15%
	Lignans, neolignans and related compounds	2	0.31%
	Lipids and lipid-like molecules	186	28.62%
	Nucleosides, nucleotides, and analogues	17	2.62%
	Organic acids and derivatives	69	10.62%
	Organic nitrogen compounds	5	0.77%
	Organic oxygen compounds	85	13.08%
	Organoheterocyclic compounds	47	7.23%
	Organooxygen compounds	1	0.15%
	Organosulfur compounds	6	0.92%
	Phenylpropanoids and polyketides	84	12.92%
	Others	126	19.38%
Class	2-arylbenzofuran flavonoids	4	0.62%
	Allyl sulfur compounds	1	0.15%
	Aurone flavonoids	1	0.15%
	Azepines	1	0.15%
	Azolidines	1	0.15%
	Benzene and substituted derivatives	9	1.38%
	Benzodioxoles	1	0.15%
	Benzopyrans	3	0.46%
	Camptothecins	1	0.15%
	Carbohydrates and carbohydrate conjugates	1	0.15%
	Carboxylic acids and derivatives	58	8.92%
	Cinnamic acids and derivatives	18	2.77%
	Coumarins and derivatives	4	0.62%
	Cyclobutane lignans	1	0.15%
	Depsides and depsidones	3	0.46%
	Diarylheptanoids	2	0.31%
	Diazinanes	1	0.15%
	Diazines	2	0.31%
	Dihydrofurans	1	0.15%
	Dihydroisoquinolines	1	0.15%
	Fatty Acyls	65	10.00%
	Flavin nucleotides	1	0.15%
	Flavonoids	31	4.77%
	Furofurans	2	0.31%
	Furopyrans	1	0.15%
	Glycerolipids	11	1.69%
	Glycerophospholipids	29	4.46%
	Heteroaromatic compounds	4	0.62%
	Hydroxy acids and derivatives	3	0.46%
	Imidazopyrimidines	2	0.31%
	Indoles and derivatives	5	0.77%
	Isocoumarans	1	0.15%
	Isoflavonoids	6	0.92%
	Kavalactones	1	0.15%
	Keto acids and derivatives	3	0.46%
	Lactones	3	0.46%
	Lignan glycosides	1	0.15%
	Linear 1,3-diarylpropanoids	5	0.77%
	Macrolides and analogues	1	0.15%
	Naphthalenes	1	0.15%
	Nucleoside and nucleotide analogues	2	0.31%

	Organic disulfides	1	0.15%
	Organic oxoanionic compounds	1	0.15%
	Organic phosphonic acids and derivatives	1	0.15%
	Organic sulfuric acids and derivatives	3	0.46%
	Organonitrogen compounds	5	0.77%
	Organooxygen compounds	84	12.92%
	Oxanes	1	0.15%
	Peptidomimetics	1	0.15%
	Phenol esters	1	0.15%
	Phenol ethers	2	0.31%
	Phenols	4	0.62%
	Phenylpropanoic acids	1	0.15%
	Piperidines	2	0.31%
	Polyketides	24	3.69%
	Prenol lipids	33	5.08%
	Pteridines and derivatives	1	0.15%
	Purine nucleosides	2	0.31%
	Purine nucleotides	9	1.38%
	Pyridine nucleotides	1	0.15%
	Pyridines and derivatives	2	0.31%
	Pyrimidine nucleosides	1	0.15%
	Pyrrolizidines	1	0.15%
	Pyrrolizines	1	0.15%
	Quinolines and derivatives	5	0.77%
	Ribonucleoside 3'-phosphates	1	0.15%
	Saxitoxins, gonyautoxins, and derivatives	1	0.15%
	Sphingolipids	7	1.08%
	Steroids and steroid derivatives	11	1.69%
	Sterol Lipids	6	0.92%
	Stilbenes	4	0.62%
	Sulfoxides	2	0.31%
	Tannins	2	0.31%
	Tetralins	1	0.15%
	Tetrapyrroles and derivatives	5	0.77%
	Thioethers	1	0.15%
	Thiols	1	0.15%
	Tropane alkaloids	1	0.15%
	Unsaturated hydrocarbons	1	0.15%
	Others	127	19.54%
Sub Class	1-benzopyrans	2	0.31%
	1-hydroxy-2-unsubstituted benzenoids	1	0.15%
	2-benzopyrans	1	0.15%
	Alcohols and polyols	9	1.38%
	Alkylthiols	1	0.15%
	Alloxazines and isoalloxazines	1	0.15%
	Amines	4	0.62%
	Amino acids, peptides, and analogues	55	8.46%
	Anisoles	1	0.15%
	Arylsulfates	2	0.31%
	Benzenediols	1	0.15%
	Benzoic acids and derivatives	4	0.62%
	Benzoquinolines	3	0.46%
	Beta hydroxy acids and derivatives	2	0.31%
	Bile acids, alcohols and derivatives	3	0.46%

Bisphosphonates	1	0.15%
Carbohydrates and carbohydrate conjugates	66	10.15%
Carbonyl compounds	6	0.92%
Chalcones and dihydrochalcones	2	0.31%
Chlorins	1	0.15%
Cinnamic acid esters	1	0.15%
Cinnamic acids	1	0.15%
Cinnamylphenols	2	0.31%
Coumarin glycosides	2	0.31%
Cyclic purine nucleotides	2	0.31%
Dialkyldisulfides	1	0.15%
Dialkylthioethers	1	0.15%
Dicarboxylic acids and derivatives	1	0.15%
Diterpenoids	4	0.62%
Eicosanoids	3	0.46%
Ethers	3	0.46%
Fatty acid esters	2	0.31%
Fatty acids and conjugates	20	3.08%
Fatty acyl glycosides	17	2.62%
Fatty acyl thioesters	3	0.46%
Fatty alcohols	3	0.46%
Fatty amides	4	0.62%
Flavones	3	0.46%
Flavonoid glycosides	26	4.00%
Flavonoids	22	3.38%
Furanocoumarins	1	0.15%
Furanoisoflavonoids	1	0.15%
Furanones	1	0.15%
Furanoquinolines	2	0.31%
Gamma butyrolactones	3	0.46%
Glycerophosphates	7	1.08%
Glycerophosphocholines	3	0.46%
Glycerophosphoglycerols	5	0.77%
Glycerophosphoinositolglycans	1	0.15%
Glycerophosphoinositols	7	1.08%
Glycerophosphoserines	2	0.31%
Glycosphingolipids	1	0.15%
Glycosyldiradylglycerols	1	0.15%
Glycosylglycerols	2	0.31%
Halobenzenes	1	0.15%
Hopanoids	1	0.15%
HyBcid peptides	1	0.15%
Hydrocarbons	2	0.31%
Hydrolyzable tannins	2	0.31%
Hydropyridines	1	0.15%
Hydroxycinnamic acids and derivatives	16	2.46%
Hydroxycoumarins	1	0.15%
Hydroxyflavonoids	1	0.15%
Hydroxyindoles	1	0.15%
Hydroxyisoflavonoids	1	0.15%
Indoles	1	0.15%
Indolines	1	0.15%
Indolyl carboxylic acids and derivatives	1	0.15%
Isoflavonoid O-glycosides	4	0.62%

Isoprenoids	1	0.15%
Isosorbides	2	0.31%
Linear diarylheptanoids	2	0.31%
Lineolic acids and derivatives	3	0.46%
Macrolides and lactone polyketides	1	0.15%
Medium-chain hydroxy acids and derivatives	1	0.15%
Medium-chain keto acids and derivatives	3	0.46%
Methoxybenzenes	1	0.15%
Methoxyphenols	2	0.31%
Monoradylglycerols	3	0.46%
Monosaccharides	1	0.15%
Monoterpenoids	1	0.15%
N-acylpiperidines	1	0.15%
Naphthalene sulfonic acids and derivatives	1	0.15%
Nicotinamide nucleotides	1	0.15%
Octadecanoids	5	0.77%
O-methylated flavonoids	1	0.15%
Organic pyrophosphates	1	0.15%
Other Fatty Acyls	2	0.31%
Other Polyketides	1	0.15%
Other Sphingolipids	3	0.46%
Oxazolidines	1	0.15%
Oxidized glycerophospholipids	4	0.62%
Oxygenated hydrocarbons	1	0.15%
Phosphosphingolipids	2	0.31%
Piperazines	1	0.15%
Polyprenols	1	0.15%
Polyprenylphenols	1	0.15%
Porphyrins	3	0.46%
Pregnane steroids	1	0.15%
Purine deoxyribonucleotides	1	0.15%
Purine ribonucleotides	6	0.92%
Purines and purine derivatives	2	0.31%
Pyridinecarboxylic acids and derivatives	1	0.15%
Pyrimidines and pyrimidine derivatives	2	0.31%
Quaternary ammonium salts	1	0.15%
Quinone and hydroquinone lipids	1	0.15%
Secosteroids	1	0.15%
Sesquiterpenoids	5	0.77%
Sphingoid bases	1	0.15%
Steroid conjugates	1	0.15%
Steroid esters	2	0.31%
Steroid lactones	2	0.31%
Steroidal glycosides	3	0.46%
Steroids	2	0.31%
Sterols	2	0.31%
Stilbene glycosides	1	0.15%
Sulfuric acid esters	1	0.15%
Terpene glycosides	10	1.54%
Terpene lactones	3	0.46%
Tetraterpenoids	3	0.46%
Tricarboxylic acids and derivatives	2	0.31%
Triradylcglycerols	4	0.62%
Triradylglycerols	1	0.15%

Triterpenoids	2	0.31%
Tryptamines and derivatives	1	0.15%
Unsaturated aliphatic hydrocarbons	1	0.15%
Others	177	27.23%

Supplementary Table S2 Classification analysis of DAMs in GC-MS/MS

	Category	Number of metabolites	Proportion (%)
Sup Class	Benzenoids	6	5.41%
	Lipids and lipid-like molecules	12	10.81%
	Nucleosides, nucleotides, and analogues	2	1.80%
	Organic acids and derivatives	30	27.03%
	Organic compounds	1	0.90%
	Organic nitrogen compounds	2	1.80%
	Organic oxygen compounds	16	14.41%
	Organoheterocyclic compounds	13	11.71%
	Organooxygen compounds	1	0.90%
	Phenylpropanoids and polyketides	4	3.60%
	Prenol lipids	1	0.90%
	Others	23	20.72%
Class	5'-deoxyribonucleosides	1	0.90%
	Azoles	1	0.90%
	Benzene and substituted derivatives	3	2.70%
	Carbohydrates and carbohydrate conjugates	1	0.90%
	Carboxylic acids and derivatives	27	24.32%
	Cinnamic acids and derivatives	1	0.90%
	Coumarins and derivatives	1	0.90%
	Diazines	2	1.80%
	Fatty Acyls	6	5.41%
	Flavonoids	1	0.90%
	Furans	1	0.90%
	Glycerolipids	2	1.80%
	Glycerophospholipids	1	0.90%
	Hydroxy acids and derivatives	1	0.90%
	Imidazopyrimidines	2	1.80%
	Indoles and derivatives	3	2.70%
	Isoprenoids	1	0.90%
	Keto acids and derivatives	1	0.90%
	Lactones	1	0.90%
	Lipids and lipid-like molecules	1	0.90%
	Organic phosphonic acids and derivatives	1	0.90%
	Organonitrogen compounds	2	1.80%
	Organooxygen compounds	16	14.41%
	Phenols	3	2.70%
	Prenol lipids	2	1.80%
	Pteridines and derivatives	1	0.90%
	Purine nucleosides	1	0.90%
	Pyridines and derivatives	2	1.80%
	Steroids and steroid derivatives	1	0.90%

	Stilbenes	1	0.90%
	Others	23	20.72%
Sub Class	5'-deoxy-5'-thionucleosides	1	0.90%
	Alcohols and polyols	3	2.70%
	Alpha-keto acids and derivatives	1	0.90%
	Amines	1	0.90%
	Amino acids, peptides, and analogues	26	23.42%
	Benzaldehydes	1	0.90%
	Benzenediols	1	0.90%
	Benzoic acids and derivatives	2	1.80%
	Bile acids, alcohols and derivatives	1	0.90%
	Bipyridines and oligopyridines	1	0.90%
	Carbohydrates and carbohydrate conjugates	13	11.71%
	Cresols	1	0.90%
	Fatty acids and conjugates	4	3.60%
	Fatty acyl glycosides	1	0.90%
	Fatty alcohols	1	0.90%
	Flavans	1	0.90%
	Furoic acid and derivatives	1	0.90%
	Gamma butyrolactones	1	0.90%
	Glycerophosphates	1	0.90%
	Glycosyl compounds	1	0.90%
	Guanidines	1	0.90%
	Hydroxycinnamic acids and derivatives	1	0.90%
	Hydroxycoumarins	1	0.90%
	Hydroxyindoles	1	0.90%
	Imidazoles	1	0.90%
	Indolyl carboxylic acids and derivatives	2	1.80%
	Medium-chain hydroxy acids and derivatives	1	0.90%
	Methoxyphenols	1	0.90%
	Monoradylglycerols	2	1.80%
	Organic phosphonic acids	1	0.90%
	Pterins and derivatives	1	0.90%
	Purines and purine derivatives	2	1.80%
	Pyrimidines and pyrimidine derivatives	2	1.80%
	Pyrrolidinylpyridines	1	0.90%
	Quinone and hydroquinone lipids	2	1.80%
	Steroids and steroid derivatives	1	0.90%
	Tricarboxylic acids and derivatives	1	0.90%
	Others	26	23.42%

Supplementary Table S3. Significantly accumulated metabolite information in OPLS-DA S-plot analysis

ID	Metabolites	Sub Class	VIP	*P*-value	log_2(FC)
6.20_335.1004n			12.2884	0.00023	1.29758
5.37_679.1509m/z	3,4,5-trihydroxy-6-[4-hydroxy-3-(3-oxoprop-1-en-1-yl) phenoxy] oxane-2-carboxylic acid	Carbohydrates and carbohydrate conjugates	19.9049	9.9E-07	0.98885
5.37_649.1406m/z	Dillenetin 5-glucoside-7-glucuronide	Flavonoids	10.2084	0.00332	0.48853
5.37_339.0718m/z	6-[4-(2-carboxyethyl)-3-hydroxyphenoxy]-3,4,5-trihydroxyoxane-2-carboxylic acid	Carbohydrates and carbohydrate conjugates	17.1108	1.9E-06	0.62342
4.99_610.1526n	Kaempferol 3-gentiobioside	Flavonoids	11.8559	0.0007	1.30963
14.76_606.2837n			21.932	0.00289	1.48172
13.57_592.2681n	Pheophorbide a	Unclassified	15.6563	7E-10	3.14196
13.08_264.2453n	9,12,15-Octadecatrien-1-ol	Fatty alcohols	11.7135	0.03014	0.25094
1.12_146.0204n			12.7812	0.0006	0.88995
0.86_637.1828m/z	Ibandronate	Bisphosphonates	11.9189	1.5E-08	1.30042
0.79_180.0634n	Beta-D-Glucose	Carbohydrates and carbohydrate conjugates	10.5812	1.3E-07	1.21866
0.79_180.0625n	D-Galactose	Carbohydrates and carbohydrate conjugates	18.2445	4.1E-09	0.94706
0.77_246.0501n	Phosphatidyl glycerol	Unclassified	11.5252	5.3E-08	2.83877
0.81_342.1160n	Trehalulose	Carbohydrates and carbohydrate conjugates	9.64825	0.0106	-0.775
5.31_279.0509m/z	Benzoyl glucuronide (Benzoic acid)	Carbohydrates and carbohydrate conjugates	8.43694	0.01267	-0.8408
0.82_342.1161n	Kojibiose	Fatty acyl glycosides	7.45007	0.01104	-1.1318
2.86_148.0526n	Cinnamic acid	Cinnamic acids	8.49892	4.6E-07	-1.2436
4.45_338.1001n	3,4,5-trihydroxy-6-{[(3E)-2-oxo-4-phenylbut-3-en-1-yl]oxy}oxane-2-carboxylic acid	Fatty acyl glycosides	7.81014	0.00115	-0.644
0.81_439.0757m/z	5'-Butyrylphosphouridine	Unclassified	11.9127	0.00015	-1.0662
0.81_404.1043m/z	Quinoline-3-carboxamides	Unclassified	14.2997	4.9E-05	-2.6307
0.61_184.0162n			13.6309	1.7E-06	-0.9892
0.77_243.0586n			7.96657	0.00029	-0.6271
0.86_494.1480n			8.83281	0.00553	-1.6897
12.95_600.4166m/z			12.424	9.4E-08	-1.0015
15.48_758.2198m/z			10.8567	0.00284	-2.9219
4.43_467.1184n			10.9264	0.00057	-1.0032

Supplementary Table S4. Throughput and quality of RNA-seq of the samples at 34 DAP and 46 DAP

ID	RawReads (M)	RawBases (G)	CleanReads (M)	CleanBases (G)	ValidBases (%)	Q30 (%)	GC (%)
34 DAPa	50.41M	7.56G	49.32M	6.77G	89.57%	92.82%	48.07%
34 DAPb	47.88M	7.18G	46.88M	6.46G	89.91%	92.86%	48.21%
34 DAPc	50.06M	7.51G	48.99M	6.74G	89.70%	92.77%	48.52%
46 DAPa	49.70M	7.45G	48.71M	6.73G	90.31%	93.06%	48.19%
46 DAPb	48.86M	7.33G	47.69M	6.59G	89.86%	92.63%	47.68%
46 DAPc	47.51M	7.13G	46.42M	6.41G	89.96%	92.76%	47.73%

Supplementary Table S5. Mapped results of the samples at 34 DAP and 46 DAP with the reference genomics

ID	Total reads	Total mapped reads	Multiple mapped	Uniquely mapped	Mapping Ratio
34 DAPa	49316962	43971462(89.16%)	1280185(2.60%)	42691277(86.57%)	89.17%
34 DAPb	46884190	42031329(89.65%)	1224721(2.61%)	40806608(87.04%)	89.65%
34 DAPc	48986418	43978071(89.78%)	1273859(2.60%)	42704212(87.18%)	89.78%
46 DAPa	48713064	43514261(89.33%)	1254984(2.58%)	42259277(86.75%)	89.33%
46 DAPb	47686316	42725553(89.60%)	1242016(2.60%)	41483537(86.99%)	89.59%
46 DAPc	46417748	41551059(89.52%)	1217566(2.62%)	40333493(86.89%)	89.51%

Supplementary Table S6. Primers used for qPCR analysis

Gene name	Primer name	Primer sequence
actin	*actin-F*	5′-TGGGTTTGCTGGTGACGAT-3′
	actin-F	5′-TGCCTAGGACGACCAACAATACT-3′
SUS	*SUS3-F*	5′-CGTTGATCAGGGCAAAGGTA-3′
	SUS3-R	5′-CTATGGCTTCCATTGCGGAT-3′
BAM1	*BAM1-F*	5′-CGATGGGGAACACTGTGAAC-3′
	BAM1-R	5′-ACCTCCCCAGTTATAAGCCC-3′
BAM3	*BAM3-F*	5′-TGACTCTGTGCCTGTCCTAA-3′
	BAM3-R	5′-TTCCCACTTGAATTTCCGCA-3′
BAM5	*BAM5-F*	5′-CTCTCTTCGCTGGAAGAACC-3′
	BAM5-R	5′-CCTCGATGTCAACAATGGCT-3′
BAM3-Like	*BAM3-like-F*	5′- TGCTGGAAGAGATCAGCAAG-3′
	BAM3-like-R	5′-CAGGGACAGAATCACATCCC-3′
SS1	*SS1-F*	5′- CTGTATCCGTCCGATCTGC-3′
	SS1-R	5′- CAGACTCTCCAGAGACGGAA-3′
BraA09g036850.3C	*BraA09g036850.3C-F*	5′-CATGCATGGCCTGAAATCCA-3′
	BraA09g036850.3C-R	5′- TCAGGTGCGTATCCATCCTC-3′
BraA01g000700.3C	*BraA01g000700.3C-F*	5′-CACTCCCTGGGATCTACGAC-3′
	BraA01g000700.3C-R	5′- ACGACCACATTGCTGTAACG-3′
SAHH2	*SAHH2-F*	5′-TCACCATCAAGCCACAGACT-3′
	SAHH2-R	5′-CGGCTAACACAATGATGCCA-3′
CHI	*CHI-F*	5′-CCACCGTCTGTCATCTCACC-3′
	CHI-R	5′-CGTTAACGGCAGCTTCATCG-3′
CHS1	*CHS1-F*	5′-CTGACACCCACCTTGACTCC-3′
	CHS1-R	5′-CCCACTTCCCTCAAGTGTCC-3′
FLS1	*FLS1-F*	5′-CGACCGGATTTAGCTTTGGG-3′
	FLS1-R	5′-TCGCCGATGTGAACAATGAC-3′
CHS3	*CHS3-F*	5′-GGGCCTTCTTCGTTGGATGA-3′
	CHS3-R	5′-GTCAAGTGCATGTGGCGTTT-3′
CEL1	*CEL1-F*	5′-AGACGCTTACTCCGACCA-3′
	CEL1-R	5′-CTGTACGCACCACGATACCTATT-3′
BraA08g002960.3C	*BraA08g002960.3C-F*	5′-TGTTGGAGCACTGTCACTTGG-3′
	BraA08g002960.3C-R	5′-ACACTTGATTATGGCGTCGG-3′
BGLU16	*BGLU16-F*	5′-AACGAGCCATTTACGGTGGT-3′
	BGLU16-R	5′-TATGGCCGACGATGTAAGGC-3′
CWINV3	*CWINV3-F*	5′-ACAGCCTCCAGCATTCAGTC-3′
	CWINV3-R	5′-GGCGACGTTTTGGACTTGAC-3′
BraA05g019040.3C	*BraA05g019040.3C-F*	5′-GTCCTCACACCTCCCAAGAA-3′
	BraA05g019040.3C-R	5′- GGGTCCAAAGATGTCGGAGA-3′
BraA05g027230.3C	*BraA05g027230.3C-F*	5′- ACCAGCGAGGTTCTCTTTGA-3′
	BraA05g027230.3C-R	5′-TGAAGAGAGGGACGTTTGCT-3′
BFRUCT3	*BFRUCT3-F*	5′-GTACCACCTCCAGGTATCGG-3′
	BFRUCT3-R	5′-CCAGTGTTTGGGACTTGGTG-3′
BraA03g023380.3C	*BraA03g023380.3C-F*	5′- ATCTCAGTTCCAAGCCCACA-3′
	BraA03g023380.3C-R	5′-CTCCCACCTGTCCGTAAAGT-3′
BGLU9	*BGLU9-F*	5′- GCTACAAGACACGCCAAGAA-3′
	BGLU9-R	5′-TGGTGACCTCTTGAGACCAG-3′
BGLU15	*BGLU15-F*	5′-AAGACGCTATCTCAGTCGGA-3′
	BGLU15-R	5′-TAAACCAGCCCAAACCTGAC-3′
BGLU47	*BGLU47-F*	5′- CGTTGTTCCAAGACTTTCGC-3′
	BGLU47-R	5′-GTGACCGGATGATGTTGTGA-3′
	ARB_05372-F	5′-GGACAAGAACGGGAAATCGG-3′
ARB_05372	*ARB_05372-R*	5′-TGATCCGATTGGCTACACGA-3′
BraA06g003260.3C	*BraA06g003260.3C-F*	5′-AATGGAGGACTGTGGTGGAG-3′
	BraA06g003260.3C-R	5′-AGCCTCCTTCAGAAGCCAAT-3′

CWINV4	*CWINV4-F*	5′-AGCTTGGACATGACACGGTA-3′
	CWINV4-R	5′-TTAAACCGTCCCAGCCATCT-3′
